# Association of fibroblast growth factor 21 with alcohol consumption and alcohol liver cirrhosis

**DOI:** 10.1007/s40211-020-00380-8

**Published:** 2020-12-16

**Authors:** Jolana Wagner-Skacel, Angela Horvath, Philipp Grande, Julian Wenninger, Franziska Matzer, Christian Fazekas, Sabrina Mörkl, Andreas Meinitzer, Vanessa Stadlbauer

**Affiliations:** 1 Department of Medical Psychology and Psychotherapy, Medical University of Graz (MUG), Auenbruggerplatz 3, 8036 Graz, Austria; 2 Division of Gastroenterology and Hepatology, Medical University of Graz (MUG), Graz, Austria; 3 Department of Child and Adolescent Psychiatry, LKH Graz II, Graz, Austria; 4 Department of Psychiatry and Psychotherapeutic Medicine, Medical University of Graz (MUG), Graz, Austria; 5 Department of Clinical and Chemical Laboratory Diagnostics, Medical University of Graz (MUG), Graz, Austria

**Keywords:** Alcohol use disorder, End stage, Liver disease, FGF21, Liver-brain axis, Alkoholkonsumstörung, Endstadium, Lebererkrankung, FGF21, Leber-Gehirn-Achse

## Abstract

**Background:**

Fibroblast growth factor 21 (FGF21) is produced in the liver and binds to different complex receptor/coreceptor systems. Besides many other processes, FGF21 regulates the intake of simple sugars and alcohol. Increased levels of FGF21 decrease harmful alcohol intake in mice. To increase our understanding on the relationship between FGF21 and alcohol intake in humans, we aimed to measure FGF21 levels in patients with alcoholic liver cirrhosis (ALC) in comparison to patients with nonalcoholic liver cirrhosis (NALC) and healthy persons based on their present alcohol consumption.

**Methods:**

Alcohol intake was verified by urinary ethyl glucuronide (uETG) levels, eating and drinking behaviour by a Food Frequency Questionnaire and FGF 21 plasma levels were determined by ELISA in 96 persons (ALC *n* = 41; NALC *n* = 34; healthy *n* = 21).

**Results:**

Both ALC and NALC patients with elevated ETG levels (≥0.5 μg/ml; indicating alcohol consumption in the last 12–72 h) showed significantly higher FGF21 plasma levels in comparison to patients with negative ETG levels. Eating behaviour did not have an impact on FGF21 plasma levels.

**Conclusions:**

Increased FGF21 levels in patients with recent alcohol consumption (verified by ETG) confirmed the first part of the liver–brain endocrine axis: alcohol consumption was associated with increased FGF21 levels. We could not confirm that elevated FGF21 levels were associated with reduced alcohol intake as a result. That points towards a pathology in this pathway, which might be caused by a malfunction of β‑Klotho or FGF receptors according to other studies and chronic alcohol dependency. Further research is required to clarify these pathologies, which may open new pharmacological treatment for patients with alcohol use disorder and alcohol dependence.

## Abstract (de)

**Grundlagen:**

Fibroblast-Growth-Factor 21 (FGF21) wird in der Leber produziert und bindet an verschiedene komplexe Rezeptor/Corezeptor-Systeme. FGF21 reguliert neben vielen anderen Prozessen die Aufnahme von einfachem Zucker und Alkohol. Erhöhte FGF21-Spiegel verringern den schädlichen Alkoholkonsum bei Mäusen. Um die Beziehung zwischen FGF21 und Alkoholkonsum beim Menschen besser zu verstehen, wurden FGF21-Spiegel bei PatientInnen mit alkoholischer Leberzirrhose (ALC) im Vergleich zu PatientInnen mit nichtalkoholischer Leberzirrhose (NALC) und gesunden Personen gemessen und in Korrelation mit ihrem gegenwärtigen Alkoholkonsum analysiert.

**Methodik:**

Der Alkoholkonsum wurde durch Ethylglukuronid(uETG)-Werte im Urin, das Ess- und Trinkverhalten durch einen Lebensmittelfrequenz-Fragebogen verifiziert, und die FGF21-Plasmaspiegel wurden mittels ELISA bei 96 Personen bestimmt (ALC *n* = 41; NALC *n* = 34; gesund *n* = 21).

**Ergebnisse:**

ALC- und NALC-PatientInnen mit erhöhten ETG-Spiegeln (≥0,5 μg/ml; entspricht Alkoholkonsum in den letzten 12–72 h) zeigten signifikant höhere FGF21-Plasmaspiegel im Vergleich zu Patienten mit negativen ETG-Spiegeln. Das Essverhalten hatte keinen Einfluss auf die FGF21-Plasmaspiegel.

**Schlussfolgerungen:**

Erhöhte FGF21-Spiegel bei Patienten mit kürzlich aufgetretenem Alkoholkonsum (durch ETG verifiziert) bestätigten den ersten Teil der endokrinen Achse von Leber und Gehirn: Alkoholkonsum ist mit erhöhten FGF21-Spiegeln verbunden. Wir konnten nicht bestätigen, dass erhöhte FGF21-Spiegel mit einem in Folge reduzierten Alkoholkonsum einhergehen. Dies deutet auf eine Pathologie auf diesem Weg hin, die durch eine Fehlfunktion der β‑Klotho- oder FGF-Rezeptoren und die chronische Alkoholkrankheit verursacht werden könnte. Weitere Forschungen sind erforderlich, um diese Pathologien zu klären, die möglicherweise eine neue pharmakologische Behandlung für Patienten mit Alkoholkonsumstörung und Alkoholabhängigkeit eröffnen.

## Introduction

Alcohol use disorder is a leading cause of morbidity and mortality [1] and refers to impaired control over alcohol use, leading to physiological dependency and tolerance with psychological, social and physical consequences. While the alcohol consumption in Austria was below average in 2010, Austrians had the highest largest rate of chronic liver disease and liver cirrhosis in Europe in 2017 [2]. Prevalence of alcohol use disorders and dependence was roughly 15% in Austria in 2014 [3, 4], proving a tremendous burden of disease and socioeconomic costs. Examination of different indicators of alcohol consumption and its possible health effects in Austria suggest that alcohol consumption behaviour is becoming more moderate, thus, decreasing the negative effects associated with alcohol [5].

Contemporary psychiatric research suggests that developmental and neurobiological pathways are related to the dopamine system, oxytocin system and the glucocorticoid system in alcohol use disorder [6]. The pathways leading to alcohol addiction are complex and multidimensional including dysregulation of molecular and gene expression, altered brain sensitivities to reward- and stress-related cues and behavioural patterns that include risk taking, social isolation or especially stress dysregulation [6]. The pathophysiology of alcohol dependency is not only a dysregulation of neuronal function; it can be understood as a systemic disease with alterations induced by various metabolic stresses. Alcohol consumption increases circulating levels of fibroblast growth factor (FGF) 21 in humans and mice [7]. FGF proteins are classified into endocrine- and paracrine/autocrine-regulated energy and are involved in a wide variety of biological metabolic processes. FGF 21 with a half-life of 0.7–1.1 h is expressed in liver, white adipose tissue, brown adipose tissue and pancreas and seems to influence drinking and eating preferences. FGF21 as a neurotropic hormone acts on the nervous system by suppressing the intake of alcohol. This provides evidence for a feedback liver–brain endocrine pathway that limits alcohol consumption [7]. The effects of FGF21 on the central nervous system (CNS) are associated with decreased dopamine, a key neurotransmitter used in reward pathways [8]. The family of fibroblast growth factors (FGFs) consists of 22 members; these proteins function as signalling molecules with endocrine or paracrine function and influence for example energy and mineral metabolism, tissue repair and organogenesis in early development [9]. FGF21 is a key regulating protein in energy metabolism; it is highly active in liver, white adipose and brown adipose tissue and the pancreas [10]. Its function is regulated by β‑Klotho, a cofactor necessary for the receptor binding of FGF21 in target organs that is expressed in several of these tissues. In addition to the expression of β‑Klotho in the CNS, FGF21 can cross the blood–brain barrier [11]. FGF21 administration reduces sweet and alcohol preference in mice, requires the FGF21 coreceptor β‑Klotho in the central nervous system and correlates with reductions in dopamine concentrations in the nucleus accumbens [8]. FGF21 also increases insulin sensitivity and leads to a decline of plasma glucose, triglycerides, insulin and glucagon [12]. In mice, supraphysiological levels of FGF21 lead to a reduced intake of alcohol, which is most likely due to changes in drinking behaviour rather than in ethanol metabolism [8]. Among its central actions, FGF21 induces corticotropin-releasing factor, suppresses arginine vasopressin expression in the hypothalamus and leads to reductions in dopamine concentrations in the nucleus accumbens [13]. Chronic alcohol consumption stimulates dopamine release from the major terminal area of the mesolimbic dopamine system, the nucleus accumbens [14]. Chronic alcohol consumption is associated with functional alterations of this important part of the brain reward system [14]. This leads to the question whether there is a liver–brain reward system.

FGF21 increases systemic glucocorticoid levels, suppresses physical activity and alters circadian behaviour [15].

To better understand the relation of FGF21 and alcohol intake in humans, we measured FGF21 levels in patients with alcoholic liver cirrhosis (ALC), in patients with nonalcoholic liver cirrhosis (NALC) and healthy persons to correlate their present alcohol consumption.

## Methods

From July 2012–September 2013, 101 patients with liver cirrhosis were screened in the outpatient clinic at the Department of Gastroenterology and Hepatology or Department of Transplantation Surgery of the Medical University Hospital Graz. Patients were subdivided by their aetiology of liver cirrhosis. One group included patients with alcoholic cirrhosis and the second group consisted of patients with all other types of cirrhosis. The healthy control group comprised 21 persons without liver disease or history of alcohol abuse. The inclusion criteria were patients aged between 18 and 80 years with clinical and radiological evidence of cirrhosis and a biopsy-proven liver cirrhosis of any cause. The study was approved by the Ethics Committee of the Medical University of Graz (23-096 ex 10/11) and informed consent was obtained from all participants.

The exclusion criteria were Child–Pugh score over 11, clinical evidence of active infection, antibiotic treatment within 7 days prior to enrolment, gastrointestinal haemorrhage within the previous 2 weeks, use of immunomodulating agents within the previous month, concomitant use of supplements (pre-, pro-, or synbiotics) likely to distort FGF21 levels, renal failure (such as hepatorenal syndrome), creatinine >1.7 mg/dL, hepatic encephalopathy II–IV, pancreatitis or other organ failure, hepatic or extrahepatic malignancy, pregnancy and presumed noncompliance to the study protocol.

A food frequency questionnaire was used to describe the nutritional behaviour of patients, as well as blood sampling to evaluate FGF21 and urine samples to measure ethyl glucuronide (ETG) and ethyl sulfate (ETS) levels. Additional information was collected during the clinical investigation, such as Child–Pugh score, MELD (model of end-stage liver disease) score and BMI (body mass index). The Child–Pugh’s classification is used to categorize disease severity based on clinical criteria like severity of ascites and hepatic encephalopathy, as well as synthetic performance, measured by albumin and prothrombin time, and excretion capacity obtained by bilirubin. This scoring system is helpful to predict prognosis and mortality and to define the strength of therapy. Another classification to categorize the severity of liver disease, especially in the end stage is the model of end-stage liver disease (MELD score).

### Food frequency questionnaire

The food frequency questionnaire of the nutritional medicine service of the University Hospital Graz includes 33 categories comprising the most common food and beverage items, for which each patients can indicate the frequency of consumption from “rarely/never” to “multiple times daily”. The questionnaire adapted from the Robert Koch Institute named “Ernährungsfragebogen” consisted of 57 questions with single choice answer options about intake frequency (e.g. “once a day”, “several times a day”, “2–3 a week”, “once a month”, “never”) of a wide range of food items, e.g. grain products, meat products, sweets and alcoholic beverages.

### FGF 21

FGF 21 levels were determined using a commercially available ready to use sandwich ELISA (enzyme linked immunosorbent assay) by Biovendor (Karasek, Czeck Republic) according to the manufacturer’s instructions.

### ETG and ETS

The ETG and ETS urine levels were measured by liquid chromatography–mass spectrometry using TSQ Quantum Discovery and Dionex Ultimate 3000 (Brno, Czech Republic).

## Statistical analysis

Statistical analysis was performed with SPSS 23 (IBM, Armonk, New York, USA). Chi-squared test/Fisher exact test was used to evaluate between-group differences of categorical variables. Furthermore, the assessment of the normal distribution of all continuous values was performed using the Kolmogorov–Smirnov test. As variables were not normally distributed, the Mann–Withney U‑test was used to evaluate significances in variables with two subgroups and the Kruskal–Wallis test was used for variables with three factor levels. Differences in FGF-21 levels for the three study groups were tested by a single factor analysis of variance (ANOVA), as this procedure has shown to be robust even when the requirements of normally distributed data are not met [16–18].

## Results

Of the 101 patients with liver cirrhosis of any aetiology screened in the outpatient clinic of the University Hospital of Graz, 9 of the patients had screening failures; thus, 92 patients were included: 17 of the patients met exclusion criteria, samples of 75 patients were used for further analysis (41 with alcoholic cirrhosis, 34 nonalcoholic cirrhosis, 21 healthy individuals, screened at the outpatient clinic of the research group). The age of the healthy control group was comparable to the age of the patient cohort. Table 1 gives an overview on sociodemographic and clinical characteristics of the study groups.

**Table 1:** Sociodemographic and clinical description of groups with ALC (*n* = 41), NALC (*n* = 34) and healthy controls (*n* = 21)

Variables	Groups	*n* (%)	Mean (SD)	Median (range)
Sex (females in %)	ALC	9 (22%)	–	–
	NALC	13 (38%)	–	–
	Controls	12 (57%)	–	–
Age	ALC	–	55.4 (9.2)	56.0 (39.0)
	NALC	–	57.9 (9.7)	60.0 (38.0)
	Controls	–	58.0 (7.0)	58.0 (23.0)
BMI	ALC	–	27.0 (4.0)	27.0 (21.7)
	NALC	–	27.2 (4.1)	26.3 (17.0)
	Controls	–	25.0 (3.1)	25.2 (12.5)
Child–Pugh score	ALC	–	6.1 (1.4)	6.0 (6.0)
	NALC	–	5.9 (1.3)	5.0 (5.0)
MELD score	ALC	–	12.5 (4.4)	12.4 (19.4)
	NALC	–	10.6 (3.4)	9.7 (11.4)

*ALC* alcoholic liver cirrhosis, *NALC* nonalcoholic liver cirrhosis, *SD* standard deviation, *BMI* body mass index, *MELD score* model of end-stage liver disease score

Alcohol consumption was determined via self-reports and ETG levels were measured as a marker of alcohol intake within the last 12–72 h (Table 2). Questions about alcohol intake in the food frequency questionnaire referred to the frequency of alcohol intake (such as beer, wine and other alcoholic beverages) per week. Self-reported alcohol intake was higher in the healthy control group (mean rank = 67.63) than in patients with alcoholic (mean rank = 41.38) or nonalcoholic (mean rank = 44.44) liver cirrhosis (Kruskal–Wallis H = 21.82, *p* < 0.001). Similarly, 88% of patients with ALC and 79% of patients with NALC reported to be abstinent, while only 33% of healthy controls reported alcohol abstinence. Concerning ETG levels, 78% of patients with ALC and 88% of patients with NALC had negative ETG levels (<0.5 μg/ml) in contrast to healthy controls, where all subjects had negative ETG levels. Table 2 gives information on the distribution of self-reported alcohol consumption and ETG levels in the study groups.

**Table 2:** Self-reported alcohol consumption and ETG levels in groups with ALC (*n* = 41), NALC (*n* = 34) and healthy controls (*n* = 21)

Variables	Groups	*n* (%)	Mean (SD)	Median (IQR)
Self-reported alcohol intake (units/week)	ALC	–	0.5 (2.2)	0.0 (0.0)
	NALC	–	0.2 (0.6)	0.0 (0.0)
	Controls	–	2.2 (2.7)	0.8 (3.0)
Self-reported abstinence	ALC	36 (88)	–	–
	NALC	27 (79)	–	–
	Controls	7 (33)	–	–
Negative ETG (<0.5 μg/ml)	ALC	32 (78)	–	–
	NALC	30 (88)	–	–
	Controls	21 (100)	–	–
Positive ETG (>0.5 μg/ml) and according ETG levels	ALC	9 (22)	22.6 (36.5)	10.0 (30.2)
	NALC	4 (12)	20.1 (24.9)	12.5 (45.1)
	Controls	0 (0)	–	–

*ALC* alcoholic liver cirrhosis, *NALC* nonalcoholic liver cirrhosis, *IQR* interquartile range, *ETG* ethyl glucuronide, *SD* standard deviation

FGF-21 levels were determined and compared in all three groups; no statistical differences were found for FGF-21 levels in patients with ALC (M = 491.6, SD = 693.2), NALC (M = 318.5, SD = 470.5) and healthy controls (M = 299.1, SD = 216.6; F (2.93) = 1.305, *p* = 0.276). FGF-21 levels were compared among subgroups with positive or negative alcohol consumption and positive or negative ETG levels for every study group. Significantly higher FGF21 levels were seen in patients with positive ETG levels compared to patients with negative ETG levels for both the ALC and the NALC group (Table 3). There were no differences in FGF-21 levels between groups with positive and negative self-reported alcohol consumption, neither in patient groups nor in healthy controls (Table 3).

**Table 3:** Levels of FGF21 (pg/ml) in groups with ALC (*n* = 41), NALC (*n* = 34) and healthy controls (*n* = 21) according to self-reported drinking behaviour and ETG levels

Groups	Alcohol consumption		*n* (%)	Mean (SD)	Median (IQR)	*p*-value (U-test)
ALC	Self-reported alcohol consumption	Positive	5 (12)	471 (624)	156 (359)	–
		Negative	36 (88)	494 (710)	275 (348)	0.5744
	ETG levels	Positive	9 (22)	1056 (1216)	589 (1056)	–
		Negative	32 (78)	333 (347)	213 (216)	0.0441*
NALC	Self-reported alcohol consumption	Positive	7 (21)	322 (293)	247 (289)	–
		Negative	27 (79)	317 (511)	183 (240)	0.5877
	ETG levels	Positive	4 (12)	490 (284)	431 (377)	–
		Negative	30 (88)	296 (489)	182 (182)	0.0263*
Controls	Self-reported alcohol consumption	Positive	13 (67)	303 (216)	271 (236)	–
		Negative	7 (33)	306 (247)	261 (329)	0.8773
	ETG levels	Positive	0 (0)	–	–	–
		Negative	21 (100)	299 (217)	261 (239)	–

*Positive self-reported alcohol consumption* 0.5 unit/week or more (1 unit alcohol was one beer (0.5 l), one glass of wine (0.25 l) or one shot (4 cl), e.g. 20 g pure alcohol/unit)

*Negative self-reported alcohol consumption* less than 0.5 unit/week

*Positive ETG level* >0.5 μg/ml

*Negative ETG level* <0.5 μg/ml

*ALC* alcoholic liver cirrhosis, *NALC* nonalcoholic liver cirrhosis, *IQR* interquartile range, *ETG* ethyl glucuronide, *SD* standard deviation, *FGF21* fibroblast growth factor 21

* significance p < 0.05

## Discussion

Increased FGF21 levels in patients with recent alcohol consumption (verified by ETG) confirmed the first part of the liver–brain endocrine axis: alcohol consumption is associated with increased FGF21 levels. The basic concept of the liver–brain endocrine axis is the self-protection of liver tissue by influencing the hypothalamus, which in turn leads to a reduction or cessation of alcohol consumption. This is achieved by the synthesis of FGF21, which passes the blood–brain barrier and affects the hypothalamus by binding to FGFR1c or 3c in presence of β‑Klotho [9]. This feedback mechanism seems to be vulnerable. Significantly higher FGF21 levels with positive ETG levels are seen in our study compared to those with negative ETG levels applying to the group of alcoholic liver cirrhosis and nonalcoholic liver cirrhosis. We could not confirm that elevated FGF21 levels were associated with decreased alcohol intake. That points towards a pathology in this pathway, which might be caused by a malfunction of β‑Klotho or FGF receptors according to other studies. β‑Klotho as an essential cofactor in the signal transduction of FGF21 and its variation in the *klb *gene (encoding β‑Klotho) is associated with abnormal alcohol consumption with genome-wide significance [19]. A distortion within this signal transduction pathway inhibiting the negative feedback loop might be one of the explanations why patients continue excessive alcohol consumption despite elevated FGF21 levels.

Alterations in the sensitivity of the receptor FGFR1c and 3c could be another flaw in the transmission of information in the liver–brain endocrine axis. A long-term drinking history with increased levels of FGF21 could lead to a receptor resistance, which starts a vicious circle with the consequence of further alcohol intake as a dysfunctional behaviour. Furthermore, individual and psychological characteristics including comorbid psychiatric disorders [20], early life stress [21], or impulsivity [22] are also risk factors associated with chronic alcohol consumption. Chronic administration of alcohol is associated with functional alterations of the important dopaminergic part of the brain reward system with harmful effects on cognitive functioning [23]. Among its central actions FGF21 with co-receptor β‑Klotho correlate with reductions in dopamine concentrations in the nucleus accumbens in mice [4].

Our findings suggest that increased alcohol consumption leads to increased FGF21 levels but no negative feedback leading to reduction of alcohol intake follows. This finding points to an abnormality or change in the transmission and processing of information. Dysfunction of the hypothalamic–pituitary–adrenal (HPA) axis has also been observed in chronic alcohol abuse related to stress [24]. The HPA axis affected by stress is controlled by complex interactions of a number of neurotransmitter systems to help the individual cope with stressful situations [25]. A normal HPA-axis response is characterized by a fast increase of corticotropin-releasing hormone (CRH), adrenal corticotropin (ACTH) and cortisol followed by an efficient return to prestress levels upon termination of the stressful challenge [26]. FGF 21 induces corticotropin-releasing factor and suppresses arginine vasopressin expression in the hypothalamus [10]. A number of studies have shown impairment in the activity of the HPA-axis response of alcohol-dependent individuals [27] as well as by short- and long-term abstinent alcoholics suggesting a persistent dysfunction of the HPA axis in alcohol-dependent individuals [27].

Our findings suggest an impairment in a liver–brain feedback mechanism in chronic alcohol abuse and demonstrate the importance of neuroendocrine pathways.

The second interesting result was the discrepancy concerning the declaration of alcohol intake. Monitoring alcohol use is an important part of the treatment of patients with ALC. Due to the uncertainty of alcohol intake declaration we used urine ETG levels, a frequently used test for alcohol intake in the last 12–72 h with a sensitivity of 76% and a specificity of 93%. ETG levels provided the opportunity to compare them with the information about alcohol consumption. In this context, a good match of high ETG levels and positive alcohol declaration is seen in the group of patients with nonalcoholic liver cirrhosis. The inconsistencies of alcohol declaration and high ETG in the group of patients with alcohol liver cirrhosis could be caused by cognitive impairment. Chronic excessive alcohol consumption induces cognitive impairment mainly affecting executive functions, and episodic memory [28]. These cognitive impairments not only determine daily management but also the efficiency of management and may additionally compromise the likelihood for abstinence. The presence of cognitive impairment therefore requires adaptation of the criteria of screening and the management of alcohol-dependent patients. Furthermore the inconsistencies of alcohol declaration and high ETG may be caused by shame or because the patient fear problems in their medical treatment in a liver clinic associated with access to liver transplantation. There is also a need for an active role of the caregiver with managing the therapy process and addressing the shame and guilt symptoms.

Alcohol use disorders complicate assessment and treatment of other medical and psychiatric problems. Standard criteria or perhaps new screening criteria for alcohol dependency—the more severe disorder—should be used to reliably identify people for whom long-term drinking causes major physiological consequences and the mentioned impairment of feedback mechanism and ability to homeostatic function.

Long-term alcohol dependency has a highly negative impact on bio-psycho-social regulation systems.

The evaluation of dysfunctional psychological, psychophysiological and neuroendocrine feedback regulation factors including FGF21 levels with ETG levels, cognitive and personality structure impairments could be a change towards a contemporary standardized alcoholism evaluation.

## Limitations

In our study, some patients declared no or low alcohol intake but still had positive ETG levels. The ETG test used has a sensitivity of 76% and a specificity of 93%. Exposure to alcohol in other forms (e.g. mouthwash, food, topical products) may also lead to a positive result. The dependency on the medical system could have influenced the statements. The cross-sectional design does not allow interpretation of the time-course of FGF21 increase and chronic alcohol consumption.

## Conclusion

Increased FGF21 levels in patients with recent alcohol consumption (verified by ETG) confirmed the first part of the liver–brain endocrine axis: alcohol consumption was associated with increased FGF21 levels. We could not confirm that elevated FGF21 levels are associated with a reduction of alcohol intake. That points towards a pathology in this pathway, a malfunction in the feedback mechanism, which might be caused by a malfunction of β‑Klotho or FGF receptors according to other studies. Long-term alcohol dependency could lead to alterations in the activity of regulation systems with changes in the transmission and processing of information on the molecular, neuroendocrine and behaviour level. Further research is required to clarify these pathologies, which may open new screening and pharmacological treatment for patients with alcohol use disorder and alcohol dependence.

## References

[1] Grant BF, Goldstein RB, Saha TD, Chou SP, Jung J, Zhang H, Hasin DS (2015). Epidemiology of DSM-5 alcohol use disorder: results from the National Epidemiologic Survey on Alcohol and Related Conditions III. Jama Psychiatry.

[2] Karlsen TH, Pimpin L, Webber L, Saxton J, Corbould E, Flood J. Hepahealth projekt report. European Association for the Study of the Liver. 2018. www.easl.eu. Accessed: November 2018.

[3] World Health Organization (2014). Global status report on alcohol and health 2014.

[4] World Health Organization Regional Office for Europe (2014). European detailed mortality database.

[5] Bachmayer S, Strizek J, Hojni M, Uhl A (2020). Handbuch Alkohol – Österreich. Band 1: Statistiken und Berechnungsgrundlagen 2019.

[6] Kim S, Kwok S, Mayes LC, Potenza MN, Rutherford HJ, Strathearn L (2017). Early adverse experience and substance addiction: dopamine, oxytocin, and glucocorticoid pathways. Ann NY Acad Sci.

[7] Song P, Zechner C, Hernandez G, Cánovas J, Xie Y, Sondhi V, Hu MC (2018). The hormone FGF21 stimulates water drinking in response to ketogenic diet and alcohol. Cell Metab.

[8] Talukdar S, Owen BM, Song P, Hernandez G, Zhang Y, Zhou Y, Bernardo B (2016). FGF21 regulates sweet and alcohol preference. Cell Metab.

[9] Beenken A, Mohammadi M (2009). The FGF family: biology, pathophysiology and therapy. Nat Rev Drug Discov.

[10] Fon Tacer K, Bookout AL, Ding X (2010). Research resource: comprehensive expression atlas of the fibroblast growth factor system in adult mouse. Mol Endocrinol.

[11] Coskun T, Bina HA, Schneider MA, Dunbar JD (2008). Fibroblast growth factor 21 corrects obesity in mice. Endocrinology.

[12] Potthoff M, Kliewer S, Mangelsdorf D (2016). Endocrine fibroblast growth factors 15/19 and 21: from feast to famine. Genes Dev.

[13] Owen BM, Bookout AL, Ding X, Lin VY, Atkin SD, Gautron L, Mangelsdorf DJ (2013). FGF21 contributes to neuroendocrine control of female reproduction. Nat Med.

[14] Bernardin F, Maheut-Bosser A, Paille F (2014). Cognitive impairments in alcohol-dependent subjects. Front Psychiatry.

[15] Zhao C, Liu Y, Xiao J, Liu L, Chen S, Mohammadi M, Feng W (2015). FGF21 mediates alcohol-induced adipose tissue lipolysis by activation of systemic release of catecholamine in mice. J Lipid Res.

[16] Glass GV, Peckham PD, Sanders JR (1972). Consequences of failure to meet assumptions underlying the fixed effects analyses of variance and covariance. Rev Educ Res.

[17] Salkind NJ (2010). Encyclopedia of research design.

[18] Pagano RR (2010). Understanding statistics in the behavioral sciences.

[19] Schumanna G, Liub C, O’Reillya P, Gaoe H, Songg P, Xu B (2016). KLB is associated with alcohol drinking, and its gene product β-Klotho is necessary for FGF21 regulation of alcohol preference. Proc Natl Acad Sci USA.

[20] Dawson DA, Grant BF, Stinson FS, Zhou Y (2005). Effectiveness of the derived Alcohol Use Disorders Identification Test (AUDIT-C) in screening for alcohol use disorders and risk drinking in the US general population. Alcohol Clin Exp Res.

[21] Enoch MA (2011). The role of early life stress as a predictor for alcohol and drug dependence. Psychopharmacology.

[22] Jentsch JD, Ashenhurst JR, Cervantes MC, James AS, Groman SM, Pennington ZT (2014). Dissecting impulsivity and its relationships to drug addictions. Ann NY Acad Sci.

[23] Bernardin F, Maheut-Bosser A, Paille F (2014). Cognitive impairments in alcohol-dependent subjects. Front Psychiatry.

[24] Dai X, Thavundayil J, Santella S, Gianoulakis C (2007). Response of the HPA-axis to alcohol and stress as a function of alcohol dependence and family history of alcoholism. Psychoneuroendocrinology.

[25] Tsigos C, Chrousos GP (2002). Hypothalamic–pituitary–adrenal axis, neuroendocrine factors and stress. J Psychosom Res.

[26] De Kloet ER, Derijk R (2004). Signaling pathways in brain involved in predisposition and pathogenesis of stress-related disease: genetic and kinetic factors affecting the MR/GR balance. Ann NY Acad Sci.

[27] Lovallo WR (2006). Cortisol secretion patterns in addiction and addiction risk. Int J Psychophysiol.

[28] Bernardin F, Maheut-Bosser A, Paille F (2014). Cognitive impairments in alcohol-dependent subjects. Front Psychiatry.

